# A Flexible Lithium-Ion-Conducting Membrane with Highly Loaded Titanium Oxide Nanoparticles to Promote Charge Transfer for Lithium–Air Battery

**DOI:** 10.3390/polym15102409

**Published:** 2023-05-22

**Authors:** Si-Han Peng, Yen-Hsiang Yu, Hsin-Chun Lu, Shingjiang Jessie Lue

**Affiliations:** 1 Department of Chemical and Materials Engineering, Chang Gung University, Guishan District, Taoyuan City 333, Taiwan; d1123003@cgu.edu.tw (S.-H.P.); sean0229530699@gmail.com (Y.-H.Y.); 2 Department of Orthopedics, Chang Gung Memorial Hospital, Linkou, Guishan District, Taoyuan City 333, Taiwan; 3 Department of Safety, Health and Environmental Engineering, Ming-Chi University of Technology, Taishan District, New Taipei City 243, Taiwan

**Keywords:** flexible lithium-ion-conducting membrane, solid polymer composite, hybrid electrolyte lithium–air battery, charge transfer, titanium dioxide nanoparticles

## Abstract

In this research, we aim to investigate a flexible composite lithium-ion-conducting membrane (FC-LICM) consisting of poly(vinylidene fluoride-co-hexafluoropropylene) (PVDF-HFP) and titanium dioxide (TiO_2_) nanoparticles with a TiO_2_-rich configuration. PVDF-HFP was selected as the host polymer owing to its chemically compatible nature with lithium metal. TiO_2_ (40–60 wt%) was incorporated into the polymer matrix, and the FC-LICM charge transfer resistance values (R_ct_) were reduced by two-thirds (from 1609 Ω to 420 Ω) at the 50 wt% TiO_2_ loading compared with the pristine PVDF-HFP. This improvement may be attributed to the electron transport properties enabled by the incorporation of semiconductive TiO_2_. After being immersed in an electrolyte, the FC-LICM also exhibited a R_ct_ that was lower by 45% (from 141 to 76 Ω), suggesting enhanced ionic transfer upon the addition of TiO_2_. The TiO_2_ nanoparticles in the FC-LICM facilitated charge transfers for both electron transfer and ionic transport. The FC-LICM incorporated at an optimal load of 50 wt% TiO_2_ was assembled into a hybrid electrolyte Li–air battery (HELAB). This battery was operated for 70 h with a cut-off capacity of 500 mAh g^−1^ in a passive air-breathing mode under an atmosphere with high humidity. A 33% reduction in the overpotential of the HELAB was observed in comparison with using the bare polymer. The present work provides a simple FC-LICM approach for use in HELABs.

## 1. Introduction

With the increasing demand for electronic devices, electric vehicles, and large-scale electrochemical storage systems, lithium-ion batteries cannot fully satisfy industrial needs, which is primarily due to their insufficient energy density [1]. Non-aqueous Li–air batteries have attracted research efforts because of their superior theoretical energy density [2]. Despite this, such batteries suffer from the flammability of the volatile organic liquid electrolyte (LE), along with the latent decomposition found in conventional lithium-ion batteries [3]. One serious problem is that the solid reaction product (Li_2_O or Li_2_O_2_) is insoluble in aprotic electrolytes, which hinders its practical development [4,5,6,7,8]. A Li–air battery with a hybrid electrolyte was therefore designed to avoid these detrimental effects [9,10,11,12].

Hybrid electrolyte Li–air batteries (HELABs) consist of an organic LE at the lithium anode side, a solid-state electrolyte (a lithium-ion conductive membrane (LICM)) in the middle to prevent intermixing, and an aqueous LE at the cathode side. HELABs have a Li/organic electrolyte/sodium super ionic conductor glass film (NASICON)/aqueous electrolyte/cathode structure. The HELAB directly addresses the aforementioned problems by mitigating the flammability and volatilization of aprotic LE, with the discharge product (LiOH) being soluble in an aqueous LE. In addition, HELABs can be operated under an ambient atmosphere with high relative humidity, thereby saving the cost of the high-purity O_2_ supply device required in aprotic Li–air batteries [13,14]. The most common oxides used for an LICM are NASICON-type materials such as Li_1+x_Al_x_Ti_2−x_(PO_4_)_3_ (LATP) and Li_1+x_Al_x_Ge_2−x_(PO_4_)_3_ (LAGP). However, due to its rigid nature, a ceramic LICM has a risk of cracking during assembly, followed by electrolyte leakage, corrosion of the lithium, and deterioration of the battery [13,14].

Recently, researchers have prepared solid polymer electrolytes (SPE) to take advantage of the synergistic properties of polymer–nanoparticle hybrid compositions [15]. The polymer matrix improves the mechanical properties, while the nanoparticles provide continuous pathways, enabling high ionic conductivity [16,17,18]. In order to achieve a large specific surface area and a drastic increase in the conductivity of the hybrid composites, nanoscale fillers are prepared via many methods, such as hydrothermal [19], in-situ sol-gel [20], and electrospinning methods [16,18]. Among them, ball-milling is a simple process for reducing the grain size of clustering nanoparticles and increasing the particles’ dispersivity [21]. The polymer electrolyte can be fabricated by dispersing the ceramic into a polymer matrix. The concentration of ceramic filler is small and typically has a low weight percentage relative to the polymer host [22,23]. An interesting concept called the “polymer-in-ceramic” configuration is attracting much attention by integrating a high concentration of ceramic nanoparticles into the polymer matrixes. For instance, Chen et al. studied poly(ethylene oxide) (PEO)-based SPE with the addition of ceramic at a concentration of 10 wt% to 80 wt%. The ionic conductivity gradually decreased with the increase in the proportion of the weight of the ceramic [24]. However, Zhang et al. prepared a flexible and free-standing SPE by integrating 75 wt% Li_1+x_Al_x_Ge_2−x_(PO_4_)_3_ into poly(Ɛ-caprolactone) (PCL) [17]. In that report, the greatest ionic conductivity (1.7 × 10^−4^ S cm^−1^) was obtained for a LAGP concentration of 75 wt%, validating the “polymer-in-ceramic” concept. Lu et al. incorporated 50 wt% Li_1+x_Al_x_Ti_2−x_(PO_4_)_3_ into a poly(vinylidene fluoride-co-hexafluoropropylene) (PVDF-HFP) polymer matrix to fabricate a flexible composite lithium-ion conducting membrane (FC-LICM) for use in HELABs with a cycling capacity of 500 mAh g^−1^ for up to 80 h [25]. The utilization of SPE with a sodium super ionic conductor glass film type of filler in electrochemical devices has resulted in complaints about the interfacial stability between the SPE and the lithium metal [26,27,28,29].

Titanium dioxide (TiO_2_) has recently been suggested for use as an SPE because it is a classic inorganic filler that is easy to process, has a non-toxic nature, and has chemical compatibility [30,31]. The electrochemical behavior of TiO_2_ has been extensively studied, including its electron transport, charge storage, and interfacial charge transfer [32]. The incorporation of TiO_2_ provides a higher Li^+^ ion transfer number owing to the enhanced interaction with the polymer and the promotion of Lewis acid–base interactions, which help establish ionic transport channels along the nanoparticles’ surface rather than through the polymer segment [33,34]. Hwang et al. [35] reported that the SPE’s electrolytic conductance increased with a reduction in the particles’ size. Sasikumar et al. [36] chose a PVDF-HFP/polyvinyl acetate (PVAc) blend of polymer as the host, and a LiTFSi salt in the EC was added to increase the conductivity. They investigated various weight ratios (from 2.5 wt% to 10 wt%) of TiO_2_ to PVDF-HFP/PVAc. The ionic conductivity progressively increased from 1.1 × 10^−3^ S cm^−1^ to 2.69 × 10^−3^ S cm^−1^ at a 7.5 wt% content and drastically dropped at 10 wt%, suggesting a percolation effect [37,38]. This result is ascribed to the dramatic decrease in the adsorption rate of the LE through the pore blockage with the further increase in the percentage of TiO_2_ [39]. TiO_2_-incorporated SPEs using a polymer-rich configuration (with a TiO_2_ content of 10% or less) are common in the literature [36,40]. 

Here, we present the fabrication of a solid electrolyte consisting of inorganic TiO_2_ nanoparticles and a PVDF-HFP polymeric matrix. PVDF-HFP was selected as the host polymer owing to its chemical compatibility with lithium metal [41,42,43,44]. We investigated a high load of TiO_2_ embedded into a polymer host to enhance ionic conductivity. We observed consistent improvements in the SPE’s electrochemical impedance as the TiO_2_ load increased from 40 wt% to 60 wt% compared with the bare polymer. The assembled HELAB cycled for up to 70 h and beyond. The overpotential could be reduced by up to 33% during cycling compared with using bare polymer. This demonstrated that TiO_2_-rich polymeric composites are effective solid electrolytes for application in HELABs. To the best of our knowledge, this is the first research using TiO_2_ as the inorganic nanoparticle in solid polymer electrolytes for application in a Li–air battery.

## 2. Experimental Section

### 2.1. Reagent

Lithium hydroxide monohydrate (LiOH·H_2_O, ≥ 99.0%), poly(vinylidene fluoride-co-hexafluoropropylene) (PVDF-HFP, M.W. ~455,000), lithium bis(trifluoromethylsulfonyl) imide (LiTFSI, 99.95%), tetraethylene glycoldimethyl ether (TEGDME, ≥99%), and commercial TiO_2_ powder (99.7%) were obtained from Sigma-Aldrich (St. Louis, MO, USA). Lithium chloride (LiCl, 99%) was purchased from Alfa Aesar (Ward Hill, MA, USA). N-methyl-2-pyrrolidone (NMP, ≥99.0%) was purchased from Macron Fine Chemicals (Radnor, PA, USA).

### 2.2. Pretreatment and Fabrication of the FC-LICM Incorporating TiO_2_

The commercial TiO_2_ powder was first wet ball-milled to optimize the distribution of particle size and the content of impurities. Ethanol was used as the medium. Figure S1 shows the effects of the powder’s weight per batch and the ball-milling time on the derived size distribution of TiO_2_. The distribution of the particles’ size was measured using laser diffraction particle size analyzers (Mastersizer 2000, Malvern Panalytical, Worcestershire, UK) with a detection range of 0.02–2000 μm. The dispersant’s refractive index was adjusted to 1.36 to align with the ethanol medium. Figure S1 shows that the size distribution of TiO_2_ had the strongest red shift with 3 g of TiO_2_ during the process and when the ball-milling time was fixed at 48 h. Three grams of TiO_2_ were ball-milled at 400 rpm for 48 h. The ball-milled TiO_2_ was then passed through a 200-mesh sieve. The sieved sample was then collected with an ethanol wash. The derived TiO_2_ powder was thoroughly dispersed to form a stable white colloidal solution. 

**Figure 1:**
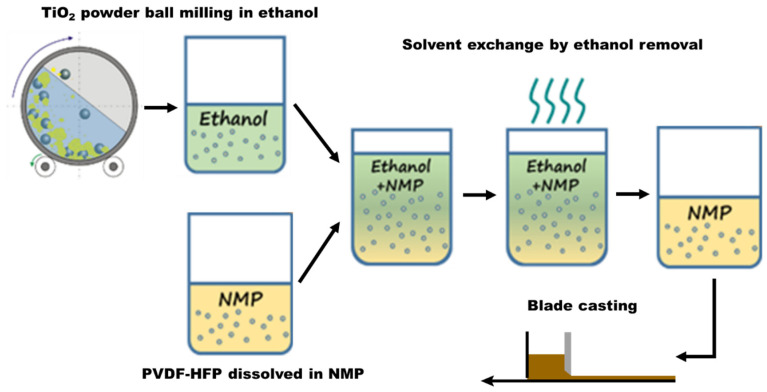
Schematic flow of preparing the flexible composite lithium-ion-conducting membrane (FC-LICM) consisting of poly(vinylidene fluoride-co-hexafluoropropylene) (PVDF-HFP) and titanium dioxide (TiO_2_) via the solvent exchange route.

The FC-LICM comprising TiO_2_ and the PVDF-HFP matrix was derived using the simple blade-casting method. First, 3 g of PVDF-HFP were dissolved in 16 g of NMP under continuous stirring at 60 °C. The slurry solution was prepared using a simple solvent exchange route, as seen in Figure 1. The TiO_2_/ethanol solution derived by ball-milling was added into the PVDF-HFP/NMP solution (with a weight ratio of TiO_2_:PVDF-HFP = 1:1) with 30 min of sonication. This solution was then stirred at 120 °C for the complete removal of ethanol. The derived slurry was then cast onto a release film (H350A, Nan Ya Plastics Corp., Taipei, Taiwan) using a film casting knife with a clearance adjusted to 500 μm. The cast film was dried in a vacuum at 60 °C for 3 days to form an FC-LICM.

### 2.3. Characterization of the Materials

The crystallinity was examined using X-ray diffraction (XRD) on a Bruker D2 Phaser (Bruker AXS GmbH, Karlsruhe, Germany) with Cu Kα radiation (λ = 1.54 Å) over the 2θ range of 10–80°. The microstructure of the as-fabricated FC-LICMs was studied using field emission scanning electron microscopy (FE-SEM, SU8000, Hitachi Ltd., Tokyo, Japan). Energy-dispersive X-ray spectroscopy (EDS) was carried out with an Xflash Detector 5030 (Bruker AXS GmbH, Karlsruhe, Germany) to determine the elemental distribution within the membrane. XPS (X-ray photoelectron spectroscopy) was performed with a K-Alpha X-ray Photoelectron Spectrometer at the R&D Center for Membrane Technology of Chung Yuan Christian University. The water permeation test was performed using a benchtop water quality meter (LAQUA F74, HORIBA, Kyoto, Japan) with a chloride ion electrode (6560S-10C). The as-fabricated FC-LICM was placed in between a glass module containing 50 mL of 2 M LiCl_(aq)_ and 50 mL of water on the other side. The chloride ion electrode was immersed on the side of the water to measure the increase in the chloride concentration. The membrane’s permeability was calculated using Equation (1) as follows:

(1) $$\mathrm{Permeability}=\frac{V}{A}\;\frac{L}{\left(C_{0}-0\right)}\;\frac{\mathrm{dC}}{\mathrm{dt}}(\frac{\mathrm{dC}}{\mathrm{dt}}>0)$$

where V is the volume of the solution, A is the cross-sectional area, L is the membrane’s thickness, and C_0_ is the initial concentration of the feed. The percentage of swelling of the membrane was measured using the ratio of the diameter before and after immersion in the aprotic electrolyte (1.0 M LiTFSI in TEGDME).

### 2.4. Electrochemical Measurements and Assembly of the Hybrid Li–Air Battery 

The charge transfer behavior of the dry FC-LICMs was measured using the AC impedance method by sandwiching the FC-LICMs between two stainless steel electrodes (with a diameter of 18 mm). The impedance was measured on an Autolab (PGSTAT302 N, Metrohm Autolab B.V., Utrecht, Netherlands) at an amplitude of 5 mV with a frequency range of 0.1 Hz to 10 MHz at room temperature. The conductivity was calculated using the following Equation (2) [40]:

(2) $$\sigma =\frac{L}{A\times R}$$

where L is the membrane’s thickness, A is the membrane’s area, and R is the resistance measured using the AC impedance test. In addition, the FC-LICMs were immersed in the aprotic electrolyte solution, and the impedance spectra were recorded on these wet FC-LICMs to investigate their charge transfer patterns.

The HELAB was constructed using CR-2032 coin cells (X2 Labwares Pte Ltd., Singapore) in a glove box (Unilab 3306-A, MBRAUN, Stratham, NH, USA). The anode side was composed of a Li metal foil (Biyuan Electronic Co., Ltd., Shenzhen, China) and a glass fiber (GF) porous separator (Whatman, Kent, UK, with a thickness of 420 μm) impregnated with 80 μL of an aprotic electrolyte (1.0 M LiTFSI in TEGDME). The as-fabricated FC-LICM was inserted between the anode and the cathode. The cathode side consisted of a commercial Pt-coated carbon cloth with a load of 5 mg cm^−2^ Pt (Yangtze Energy Technologies, Inc., New Taipei, Taiwan) and a polypropylene (PP) porous separator (Nippon Kodoshi Corp., Kochi, Japan, with a thickness of 100 μm) impregnated with 30 μL of an aqueous catholyte (an aqueous solution of 1.0 M LiOH). The coin cells were cycled on a Model BAT-750B (AcuTech System Co., Ltd., New Taipei City, Taiwan) between 2.0 V and 4.5 V at a current density of 0.05 mA cm^−2^ for 10 h per cycle when fed with ambient air (70–100% relative humidity) with a passive air-breathing mode. 

## 3. Results and Discussion

### 3.1. Characterization of TiO_2_ Nanoparticle-Filled FC-LICMs

**Figure 2:**
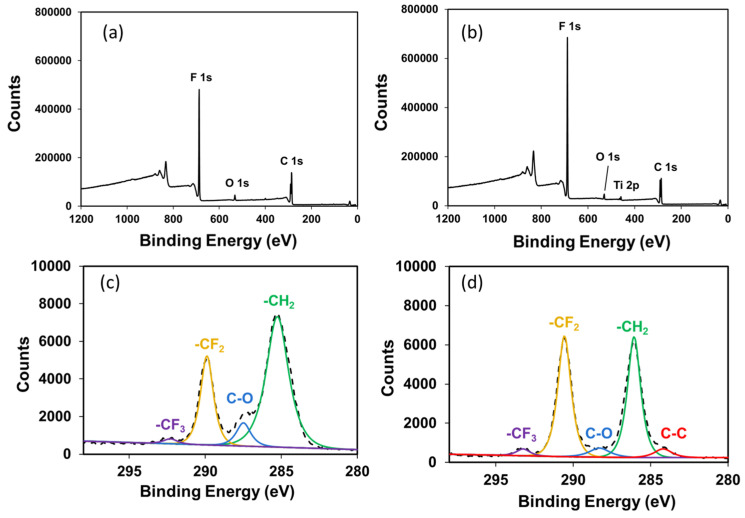
(**a**) XPS survey of the Al KR photoelectron spectra of (**a**) 0 wt% and (**b**) 50 wt% TiO_2_ incorporated into the FC-LICM sample. High-resolution XPS C 1s peak spectra of (**c**) 0 wt% and (**d**) 50 wt%.

The effects of the ball-milling treatment were studied by measuring the size of the TiO_2_ using laser diffraction particle size analyzers. As seen in Figure S2, the size distribution of the TiO_2_ was reduced from 8 μm to 90 nm on average. The broad peak around 23° in the XRD pattern was assigned to the background signal of the tape. The crystalline phase of TiO_2_ was ascribed to anatase crystals in the XRD analyses [45]. The size of the crystallite of the milled TiO_2_ was calculated to be 13.6 nm using Scherrer’s formula. The larger size distribution, as shown by the light-scattering measurements (Figure S1), resulted from the aggregated TiO_2_ nanoparticles. Figure 2 shows the XPS spectrum of the as-prepared FC-LICM containing 50 wt% TiO_2_ nanoparticles. The 2p and O 1s peaks of Ti in addition to the C 1s and F 1s signals are present, indicating that the added TiO_2_ was successfully embedded into the polymer matrix. The Ti 2p_1/2_ and 2p_3/2_ peaks are shown in Figure S3. 

The O 1s signal was observed as well, with the binding energy being the same as that of the commercial TiO_2_ powder. Note that the O 1s signal indicated a shoulder located toward higher binding energy, as seen in the commercial TiO_2_ powder. The secondary peak was assigned to hydroxyl (OH^−^) species according to the literature [46]. The peak fitting of C 1s is given in Figure 2. The spectrum has five main peaks at around 284, 285, 287, 290, and 292 eV, which were well defined [47,48,49]. As can be seen, the blending of TiO_2_ led to the spectra shifting to a higher binding energy, which was ascribed to the effect of TiO_2_ interacting with the polymer matrix. Table 1 shows a comparison of the ratio of the peak area between the -CH_2_ (predominantly influenced by vinylidene fluoride) and the -CF_2_ (predominantly influenced by hexafluoropropylene). The -CH_2_ became less prominent as the vinylidene fluoride segments were bonded to the embedded TiO_2_.

XRD and SEM analyses were carried out on the prepared FC-LICMs containing 40 wt%, 50 wt%, 60 wt% TiO_2_ to identify the crystalline and morphological properties. Well-defined crystalline XRD patterns correlating to the standard database (TiO_2_, JCPDS PDF#021-1273) were observed for each proportion of TiO_2_ (as shown in Figure S4). More importantly, the broader PVDF-HFP peak largely diminished in the FC-LICMs containing TiO_2_. This indicated that the addition of TiO_2_ hindered the alignment of the polymer chain and led to a further decrease in the degree of crystallinity. According to Fahmi et al. [50], the appearance of an amorphous region or a reduction in the crystalline region can produce high ionic conductivity. The measured thermal stability and mechanical properties are shown in Figure S5. An early decomposition of the polymer around 350 °C was observed compared with that of the bare polymer (450 °C) in our thermal stability tests. This certified the reduced crystallization with the addition of TiO_2_. The FC-LICM with 50 wt% TiO_2_ displayed a similar Young’s modulus (295 MPa) to that of bare polymer (318 MPa). 

**Figure 3:**
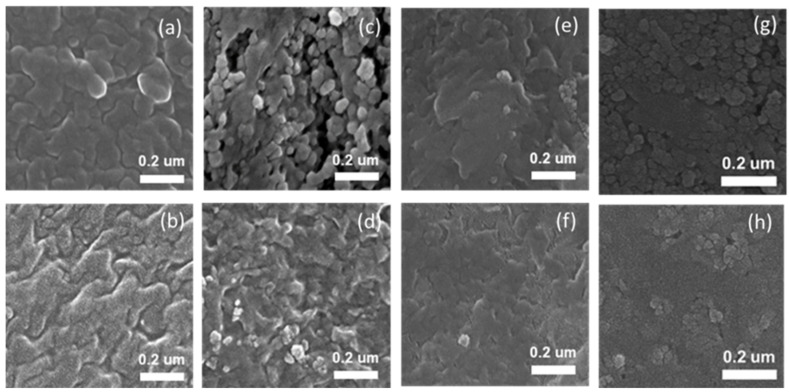
SEM images (100 k) of the front and back side of the tape-cast FC-LICM with different proportions of TiO_2_: (**a**,**b**) 0 wt% TiO_2_, (**c**,**d**) 40 wt% TiO_2_, (**e**,**f**) 50 wt% TiO_2_, and (**g**,**h**) 60 wt% TiO_2_.

Figure 3 shows the SEM images of the front (top images) and back (bottom images) of the tape-cast FC-LICM. The front side of the FC-LICM had a less dense surface with more pores in comparison with the back. A higher rate of bubble evaporation on the top resulted in the formation of pores during drying. This can best be seen in the FC-LICM with the incorporation of 40 wt% TiO_2_, for which more pores formed in comparison with the bare polymer. The voids help with the retention of the electrolyte, leading to higher conductivity [51]. 

**Figure 4:**
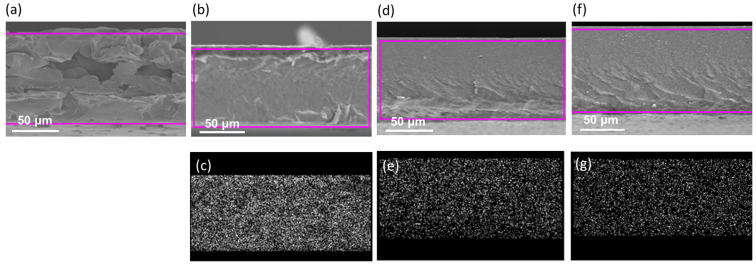
SEM cross-sectional images and EDS Ti mapping results of FC-LICMs containing (**a**) 0 wt% TiO_2_, (**b**,**c**) 40 wt% TiO_2_, (**d**,**e**) 50 wt% TiO_2_, and (**f**,**g**) 60 wt% TiO_2_.

Figure 4 shows the cross-sectional SEM images. EDS analyses were applied to display the distribution of Ti elements. It can clearly be seen that the Ti elements were well distributed across the cross-section, showing no partial deposition. Furthermore, a clearly rough cross-sectional morphology was observed in the pure polymer, which confirmed the reports in the literature [52]. Note that the cross-section micrograph changed entirely and became smooth with the addition of the nanoparticles, indicating a reduction in the crystallinity. Fahmi et al. reported that the reduction in crystallinity was caused by the distribution of random nanoparticles, which may introduce disorder in the electrolyte [50]. Thus, an increase in the conductivity of fabricated polymers with TiO_2_ nanoparticles was supported by the XRD results. 

### 3.2. Swelling Behavior and Permeating Properties of the As-Fabricated TiO_2_-Filled FC-LICMs

Permeation tests and tests of the swelling behavior were executed to identify the addition of TiO_2_ and the effects of different proportions of TiO_2_ on the prepared FC-LICMs. As shown in Table 2, the thicknesses were 94 μm, 84 μm, 86 μm, and 90 μm for FC-LICMs containing 0 wt%, 40 wt%, 50 wt%, and 60 wt% TiO_2_, respectively. The results of the permeability test indicated that the FC-LICM without TiO_2_ was the most waterproof among the candidates. The addition of TiO_2_ decreased the membranes’ compactness, allowing water to pass through in a short period of time. Among the TiO_2_-containing samples, the slowest permeability appeared at 50 wt% TiO_2_. When the TiO_2_ content was increased to 60 wt%, more pores were produced as the largest permeability was observed at this concentration. According to these results, the TiO_2_ content in the FC-LICMs is critical. 

Moreover, the addition of TiO_2_ mitigated the adsorption of the electrolyte and suppressed aprotic uptake after the FC-LICM was immersed into the aprotic electrolyte (1 M LiTFSI in TEGDME). Interestingly, these findings were not in line with the previous studies [36,51], in which composites exhibited enhanced electrolyte uptake upon the addition of filler. The swelling ratio and electrolyte uptake of the FC-LICM decreased under loading with TiO_2_.

### 3.3. Electrochemical Impedance Spectroscopy Analyses of FC-LICMs Filled with TiO_2_

**Figure 5:**
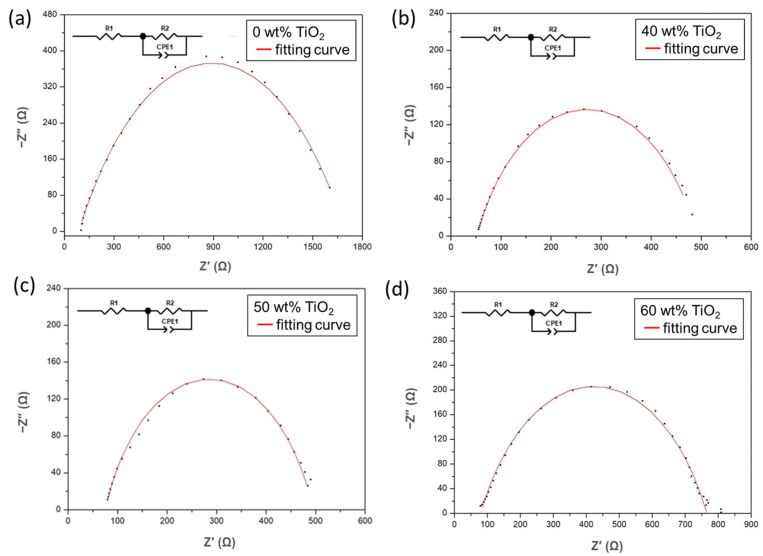
Nyquist plots of FC-LICMs with (**a**) 0 wt% TiO_2_, (**b**) 40 wt% TiO_2_, (**c**) 50 wt% TiO_2_, and (**d**) 60 wt% TiO_2_ in a dry state.

EIS was performed to analyze the charge transfer ability of the as-prepared FC-LICMs in a dry state. A Nyquist plot for each FC-LICM is shown in Figure 5. An equivalent circuit model was developed to fit the data, and the results are shown in the red curves in Figure 5. The lower intercept of the semicircle, denoted R_b_, is contributed by the bulk resistance of the dry FC-LICM, as the contribution to the impedance of the external electrical wires was negligible. Significant decreases in R_b_ were observed for the FC-LICM containing 40–60 wt% TiO_2_. The reduced R_b_ can be ascribed to the semiconductive TiO_2_ nanoparticles, which are more conductive compared with the bare polymer. 

The diameter of the semicircle, denoted R_ct_, is associated with the charge transfer impedance of the sample [53]. As depicted in Figure 5, the radius of the arc of the impedance of the dry FC-LICMs was greatly reduced in the presence of a high load of TiO_2_. The R_ct_ of the FC-LICM with 40 wt% TiO_2_ was 70.3% less than that of the bare polymer (476 vs. 1609 Ω). The most effective improvement in R_ct_ was obtained for the FC-LICM containing 50 wt% TiO_2_, with a 75.1% reduction in the R_ct_ (from 1609 Ω to 420 Ω). However, the addition of excessive TiO_2_ (60 wt%) did not reduce the R_ct_ any further, possibly due to aggregation of the nanoparticles and a loss of the effective area for the electron charge transfer. Sasikumar et al. [36] reported that a high percentage of TiO_2_ would reach a percolation threshold and cause aggregation of the particles, blocking the interfacial pathway of TiO_2_/electrolyte and suppressing the charge transfer. This finding demonstrates that the TiO_2_-loaded FC-LICMs possessed lower electron transport resistance and higher electron mobility. This result is in line with the literature [54], in which TiO_2_ was reported to serve as an important “charge transfer bridge”. The composites with a high nanofiller loading provided sufficient interconnected pathways on the surfaces of the semiconductive TiO_2_ particles for facilitating electron transport.

**Figure 6:**
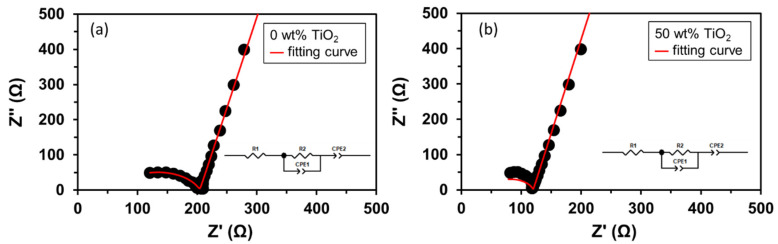
Nyquist plots of organic electrolyte-immersed FC-LICMs containing (**a**) 0 wt% TiO_2_ and (**b**) 50 wt% TiO_2_.

To assemble the HELABs, the FC-LICM was used as a separator between the aprotic TEGDME electrolyte and the aqueous LiOH solution. The FC-LICM was immersed in an aprotic electrolyte and then characterized using impedance analysis. The spectra are shown in Figure 6, with the equivalent circuit models [55,56] shown in the insets. The first real impedance intercept below 100 Ω was denoted as R_1_ and the second as R_2_. Both R_1_ and R_2_ values were several tens to several hundreds of ohms, much lower than the R_b_ and R_ct_ data in Table 3 (i.e., in the dry state) due to the presence of the lithium salt and the solvent. The FC-LICM containing 50 wt% TiO_2_ demonstrated a reduction in R_1_ from 63 to 43 Ω (a 32% decrease) compared with that without TiO_2_. The enhancement of R_1_ in the TiO_2_ composite can be ascribed to the increased amorphous phase region, as the TiO_2_ nanofillers hindered the alignment and crystallization of the polymer chain. The electrolyte-immersed FC-LCIM with 50% TiO_2_ also indicated a decrease in R_2_ from 141 to 76 Ω (45% less). The reduction in R_2_ reflected the transport of additional lithium ions. The semiconductive TiO_2_ nanoparticles served as a charge transfer bridge for transporting ions across the interface. The consistent results from Figure 5 and Figure 6 implied that the TiO_2_ nanoparticles in the FC-LICM facilitated the charge transfer for both electron transfer and ionic transport. 

### 3.4. Cycling Performance of HELABs with Different Ratios of TiO_2_-Filled FC-LICMs

**Figure 7:**
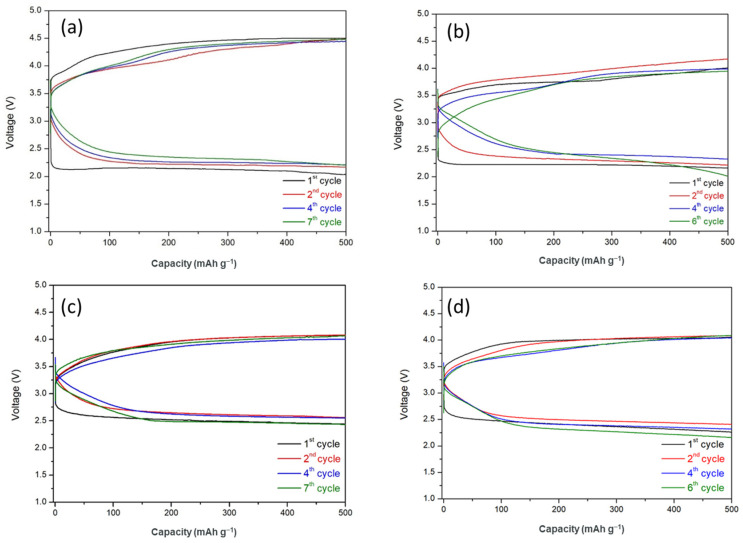
Long-term discharge/charge voltage profile of hybrid electrolyte Li–air batteries (HELABs) with FC-LICMs containing (**a**) 0 wt%, (**b**) 40 wt%, (**c**) 50 wt%, and (**d**) 60 wt% TiO_2_ under a constant current density of 0.05 mA cm^−2^ (with the charge and discharge capacities limited to 500 mAh g^−1^).

The battery performance of FC-LICMs incorporating TiO_2_ was evaluated through an assembly of the HELAB and long-term discharge/charge testing. Figure 7 shows the cycling performance of HELABs with 0 wt%, 40 wt%, 50 wt%, and 60 wt% of TiO_2_-incorporated FC-LICMs. The HELAB using the TiO_2_-free FC-LICM showed the highest overpotential (2.33 V on average), indicating the most severe electrochemical resistance. This is in line with the inferior charge transfer shown in Table 3 and Figure 5 and Figure 6. In contrast, the average overpotential was reduced to 1.85 V, 1.56 V, and 1.78 V for the HELABs with 40 wt%, 50 wt%, and 60 wt% TiO_2_ filled FC-LICM, respectively. These values indicate reductions in the over-potential of 20.6%, 33%, and 23.6% compared with the bare FC-LICM. The presence of TiO_2_ enhanced the interfacial charge transfer properties in the FC-LICM. The FC-LICM with 50 wt% TiO_2_ demonstrated the highest charge transfer rate and the least overpotential in the HELAB cycling test.

**Figure 8:**
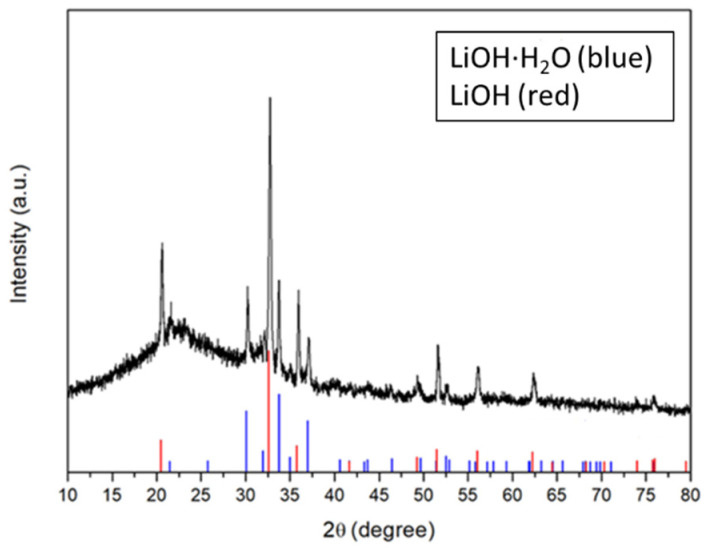
XRD pattern of the aged lithium anode after cycling of the HELAB.

Regarding the battery’s lifetime, the FC-LICM containing 50 wt% TiO_2_ displayed the longest cycle with a capacity of 500 mAh and 100% capacity retention for up to seven cycles (70 h), which was the same as for the FC-LICM without TiO_2_ but with clearly higher round-trip efficiency. Earlier deterioration was found for the batteries with 40 wt% and 60 wt% TiO_2_. Deterioration occurred after six cycles (60 h) with a drastic decline in capacity. As discussed in Section 3.2, 50 wt% TiO_2_ was more waterproof than 40 wt% TiO_2_ and 60 wt% TiO_2_, meaning slower penetration of the LE. The 50 wt% TiO_2_ proportion was more lasting because of the desired impenetrability of the FC-LICM. Overall, the failure of the battery should be ascribed to the crossover of the aqueous electrolyte and the eventual corrosion of the Li metal, which was irreversible. As seen in Figure 8, the Li metal was taken out of the dead HELABs and analyzed by XRD. The results showed that the Li metal was entirely corroded into LiOH compared with the active Li (Figure S6). The future challenge is to improve the membrane density of FC-LICM incorporating TiO_2_ to achieve greater numbers of cycles.

Due to the appropriate addition of 50 wt% TiO_2_, the assembled HELAB showed a 33% reduction in overpotential than that without TiO_2_ and provided the highest number of cycles among the FC-LICMs with various TiO_2_ loads. The higher cycling efficiency and reversibility without a clear capacity loss of the FC-LICM fabricated with 50 wt% TiO_2_ can be attributed to its higher charge transfer rate, the stable compatibility at the polymer–TiO_2_ interface, and the desired water permeation barrier. Most encouragingly, our HELAB made from FC-LICM containing 50 wt% TiO_2_ demonstrated comparable numbers of cycles and lower overpotential (a reduction of 23% from 2.04 V to 1.56 V) than the HELAB with the NASICON-based FC-LICM [25] under the same operating conditions. We suggest that an extra charge transfer path formed on the surface of the added TiO_2_ nanoparticles, resulting in a significant increase in the capacity for charge transfer.

## 4. Conclusions

This work demonstrated the preparation of a PVDF-HFP polymer with a high load semiconductive TiO_2_ to form an elective electrolyte and separator in a hybrid electrolyte lithium–air battery (HELAB). A facile approach was used using a simple casting method and a solvent exchange process, followed by the ball-milling of the TiO_2_ particles. A lower degree of polymer crystallinity was obtained by incorporating the TiO_2_ nanoparticles. The presence of a high load of TiO_2_ in the dry FC-LICM effectively promoted electron transport, as shown by the significant reduction in R_ct_ (from 1609 Ω to 420 Ω) in comparison with the pristine PVDF-HFP. The FC-LICMs incorporating TiO_2_ after infiltration of the aprotic electrolyte also exhibited a decrease in the charge transfer resistance of 45% compared with the bare polymer. These findings confirmed that the TiO_2_ nanoparticles facilitated charge transfer for both electron transfer and ionic transport. The FC-LICM containing 50 wt% TiO_2_ reduced the overpotential of the HELAB by 33% and was capable of cycling at 500 mAh beyond 70 h in a passive air-breathing mode under an atmosphere with high humidity.

## Figures and Tables

**Table 1:** Binding energy (B.E.) values and ratio of the peak area of -CH_2_- to -CF_2_- in the high-resolution C 1s spectra of FC-LICM.

Sample Name	B.E. (-CH_2_-)	B.E. (-CF_2_-)	Peak Area Ratio (-CH_2_- to -CF_2_-)
0 wt% of TiO_2_ in FC-LICM	285.2 eV	289.8 eV	2.54
50 wt% of TiO_2_ in FC-LICM	286 eV	290.5 eV	1

**Table 2:** Permeation and swelling tests for the FC-LICMs with 0 wt% TiO_2_, 40 wt% TiO_2_, 50 wt% TiO_2_, and 60 wt% TiO_2_.

TiO_2_ Content in FC-LICM	Thickness (μm)	Permeability (cm^2^ s^−1^)	Swelling Ratio (%) ^1^	Aprotic Electrolyte Uptake (%) ^2^
0 wt%	94	1.23 × 10^−9^	8.5%	13.2%
40 wt%	84	5.12 × 10^−7^	7.1%	11.8%
50 wt%	86	3.91 × 10^−7^	4.7%	5.7%
60 wt%	90	1.13 × 10^−6^	3.3%	2.7%

^1^ Increase in thickness before and after immersion in the electrolyte. ^2^ Percentage of the membrane’s weight before and after immersion in the electrolyte.

**Table 3:** Impedance and conductivity results for the FC-LICMs with 0 wt% TiO_2_, 40 wt% TiO_2_, 50 wt% TiO_2_, and 60 wt% TiO_2_ in a dry state.

TiO_2_ Content in FC-LICM	R_b_ (Ω)	R_ct_ (Ω)	Conductivity (S cm^−1^)
0 wt%	85	1609	4.31 × 10^−5^
40 wt%	48	476	6.86 × 10^−5^
50 wt%	74	420	4.54 × 10^−5^
60 wt%	79	685	4.43 × 10^−5^

## Data Availability

Not applicable.

## Supplementary Materials

The supporting information can be downloaded at: https://www.mdpi.com/article/10.3390/polym15102409/s1. Figure S1. Ball milling conditions effects on prepared TiO_2_ powder: (a) powder weight (b) ball milling time; Figure S2. TiO_2_ powder refinement before-and-after 48 hours ball milling treatment: (a) powder size distribution (b) XRD result; Figure S3. High-resolution XPS spectrum of commercial TiO_2_ powder:(a) Ti 2p peaks and (b) O 1s peak; and as-prepared 50 wt% TiO_2_ FC-LICM of (c) Ti 2p and (d) O 1s; Figure S4. XRD result of 0 wt% TiO_2_ and 50 wt% TiO_2_ incorporated FC-LICM sample; Figure S5. (a) TGA result and (b) tensile result for 0 wt% and 50 wt% TiO_2_ incorporated FC-LICM samples; Figure S6. XRD pattern of the pristine lithium anode.
