# Supplementary material for: A Flexible Lithium-Ion-Conducting Membrane with Highly Loaded Titanium Oxide Nanoparticles to Promote Charge Transfer for Lithium–Air Battery
Source: Polymers (Basel). 2023 May 22;15(10):2409. doi: 10.3390/polym15102409 (PMC10222891; doi:10.3390/polym15102409)
Supplement: Supplementary file 1 [file polymers-15-02409-s001.zip › polymers-2320071-supplementary final.pdf]

polymers

MDPI

Supplementary Materials

Article

A Flexible Lithium-Ion-Conducting Membrane with Highly Loaded Titanium Oxide Nanoparticles to Promote Charge Transfer for Lithium–Air Battery

Si-Han Peng [1], Yen-Hsiang Yu [1], Hsin-Chun Lu [1,*] and Shingjiang Jessie Lue [1,2,3,*]

[1] Department of Chemical and Materials Engineering, Chang Gung University, Guishan District, Taoyuan City 333, Taiwan; d1123003@cgu.edu.tw (S.-H.P.); sean0229530699@gmail.com (Y.-H.Y.)
[2] Department of Orthopedics, Chang Gung Memorial Hospital, Linkou, Guishan District, Taoyuan City 333, Taiwan
[3] Department of Safety, Health and Environmental Engineering, Ming-Chi University of Technology, Taishan District, New Taipei City 243, Taiwan
* Correspondence: hsinchun@mail.cgu.edu.tw (H.-C.L.); jessie@mail.cgu.edu.tw (S.J.L.)

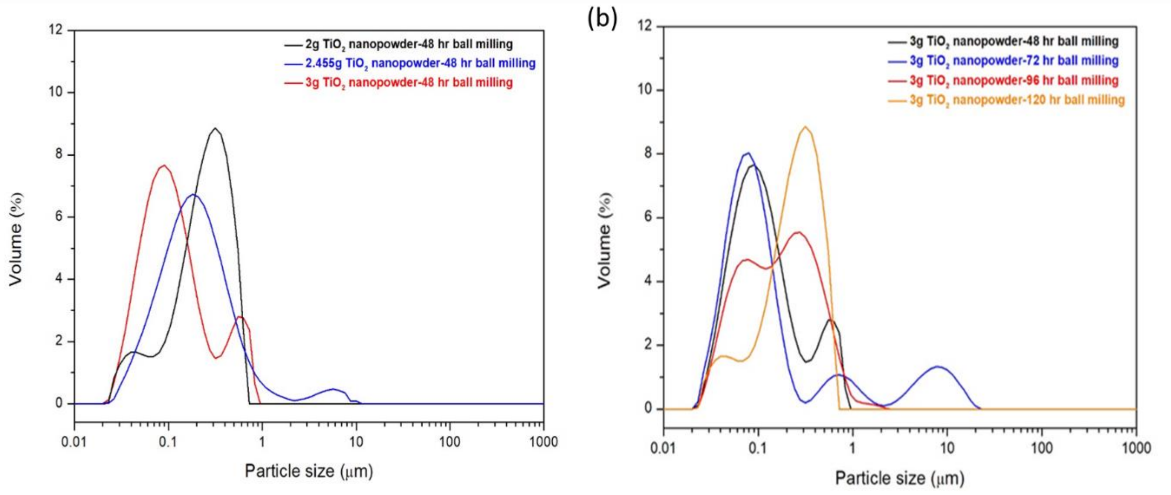

Figure S1. Ball milling conditions effects on prepared TiO_2 powder: (a) powder weight (b) ball milling time.

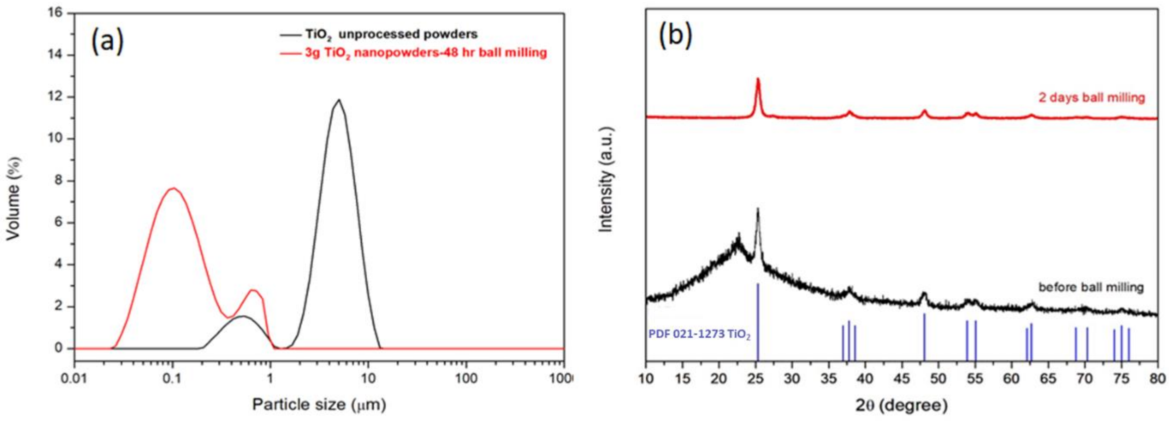

Figure S2. TiO_2 powder refinement before-and-after 48 hours ball milling treatment: (a) powder size distribution (b) XRD result.

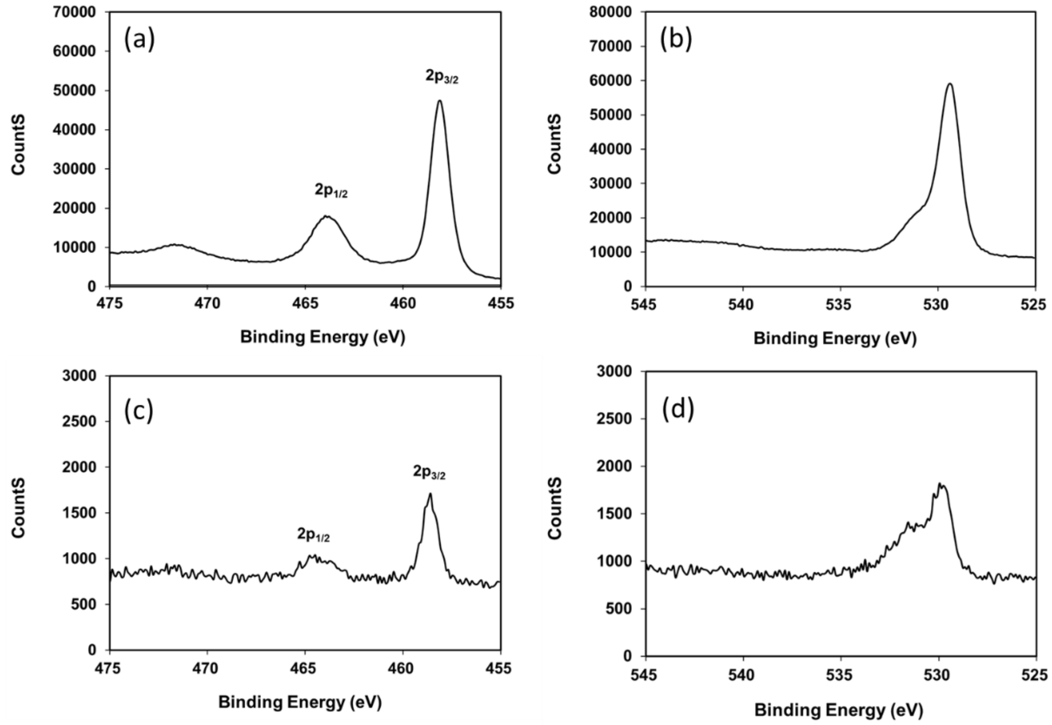

Figure S3. High-resolution XPS spectrum of commercial TiO_2 powder:(a) Ti 2p peaks and (b) O 1s peak; and as-prepared 50 wt% TiO_2 FC-LICM of (c) Ti 2p and (d) O 1s.

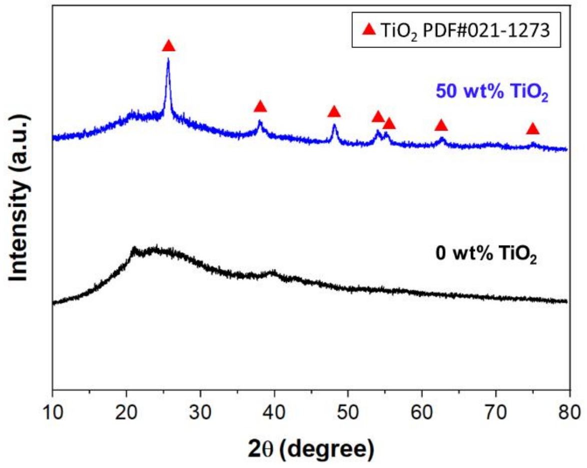

Figure S4. XRD result of 0 wt% TiO_2 and 50 wt% TiO_2 incorporated FC-LICM sample.

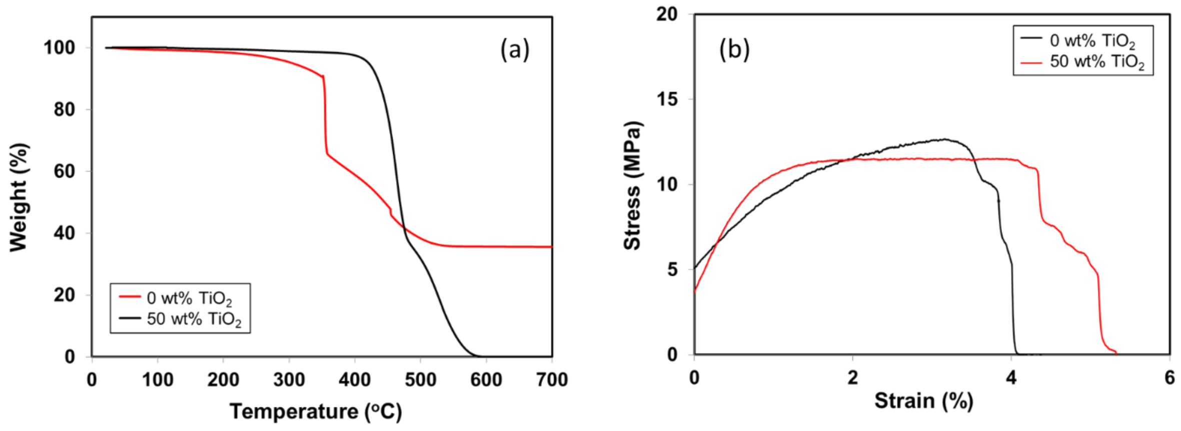

Figure S5. (a) TGA result and (b) tensile result for 0 wt% and 50 wt% TiO_2 incorporated FC-LICM samples.

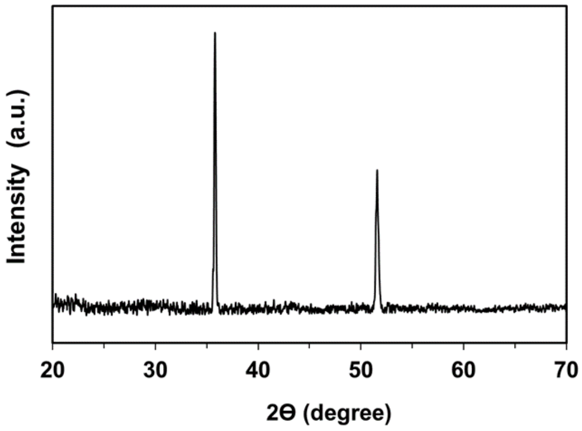

Figure S6. XRD pattern of the pristine lithium anode.
